# Lessons Learned from Post–COVID-19 Vaccination PET/CT Studies

**DOI:** 10.2967/jnumed.121.262348

**Published:** 2022-03

**Authors:** Marina Orevi, Alexandre Chicheportiche, Simona Ben Haim

**Affiliations:** 1 Department of Nuclear Medicine and Biophysics, Hadassah Medical Organization, Jerusalem, Israel;; 2 Faculty of Medicine, Hebrew University of Jerusalem, Jerusalem, Israel; and; 3 Institute of Nuclear Medicine, University College London and UCL Hospitals, NHS Trust, London, United Kingdom

**Keywords:** COVID-19, vaccination, PET/CT, lymphadenopathy, pattern

## Abstract

Vaccination against coronavirus 2019 (COVID-19) has created new challenges. Lymphadenopathy with increased uptake in patients undergoing PET/CT may mislead to unnecessary further evaluation. We have analyzed routinely performed PET/CT studies after Pfizer-BioNTech vaccination to familiarize ourselves with the PET/CT appearance of various PET tracers and to prevent the consequences of misinterpretation. **Methods:** We analyzed 1,018 PET/CT studies performed between January 1, 2021, and February 15, 2021. Information about the dates and sites of vaccination was collected. Visual and semiquantitative analysis of axillary–neck lymphadenopathy and arm uptake was correlated with immunization data. **Results:** Increased uptake in axillary lymphadenopathy was observed unilaterally in 66% of vaccinated patients, in 55% of patients vaccinated once, and in 69% of those vaccinated twice. The intensity of uptake decreased over time. Fifty-four of 274 patients (20%) had simultaneous increased activity in the posterior arm and ipsilateral axillary lymphadenopathy (double sign [DS]). The sensitivity, specificity, positive predictive value, and negative predictive value were 55.4%, 83.6%, 86.7%, 49.2%, respectively, for axillary lymphadenopathy and 38.6%, 100%, 100%, and 66.1%, respectively, for DS. No DS was observed later than 10 and 21 d after the first and the second vaccinations, respectively. None of the nonvaccinated patients had arm uptake or DS. **Conclusion:** Vaccination against COVID-19 frequently causes nonspecific axillary lymphadenopathy with increased PET tracer activity. In one fifth of our study population, this lymphadenopathy was associated with increased uptake at the vaccination site, DS. DS was 100% specific, with a 100% positive predictive value for postvaccination lymphadenopathy, hence enabling avoidance of misinterpretation of PET/CT studies and further unnecessary evaluation.

## Visual Abstract

**Figure:**
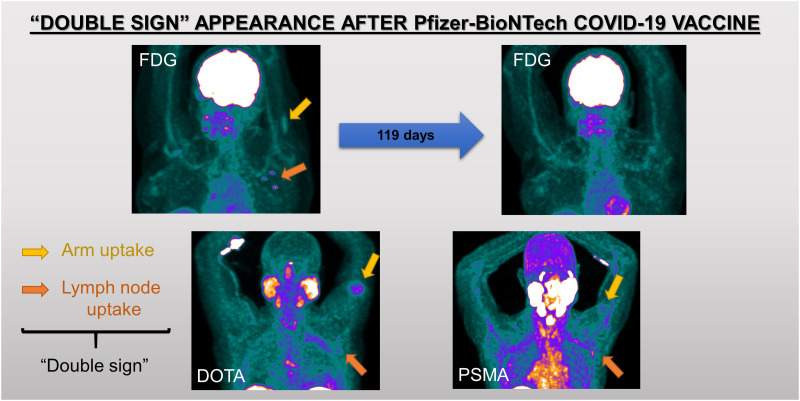

The coronavirus 2019 (COVID-19) pandemic has changed our personal and professional life (*1*). It has affected the workflow in nuclear medicine departments (*2*). Imaging findings of COVID-19 in the acute or residual stages of the disease have been detected on PET/CT (*3*,*4*).

Since December 2020, mass vaccination against COVID-19 has taken place, starting in Israel, which became one of the first countries with a high percentage of the population vaccinated. Cancer patients, routinely referred for PET/CT studies, comprise a significant subgroup of vaccinated individuals.

Lymph nodes (LNs) that house T, B, and antigen-presenting cells have an important role in the immune response to vaccination. Once injected into the muscle, the vaccine is transported to the regional LNs, and in some cases it may proceed to the next nearest lymphatic chain stations, with further activation of the T and B cells in these LNs (*5*). Incidental lymphadenopathy on physical examination, mammography, breast MRI, or PET/CT challenged interpretation of these studies (*6*–*8*). Several studies recently published demonstrated increased ^18^F-FDG uptake in post–COVID-19 vaccination lymphadenopathy (*9*–*12*).

The aim of present study was to describe the characteristics and distinctive features of the post–COVID-19 vaccination PET/CT studies with routinely used PET tracers in order to improve the confidence of PET/CT readers and to prevent unnecessary diagnostic and interventional procedures.

## MATERIALS AND METHODS

### Study Design and Participants

This retrospective study was approved by the institutional ethics committee. The need for informed consent was waived.

All PET/CT studies performed at our center between January 1, 2021, and February 15, 2021, were included. Data collected from PET/CT studies were compared with vaccination-related information and analyzed.

### PET/CT Studies

Studies were performed on either a Discovery MI digital PET/CT device or an MI-DR PET/CT device (GE Healthcare) 67 ± 9 min after injection of a 2.96 MBq/kg dose of ^18^F-FDG (*n* = 973), ^68^Ga-DOTATATE (*n* = 14), ^68^Ga-prostate-specific membrane antigen (PSMA) (*n* = 26), or ^18^F-PSMA (*n* = 5). The studies were performed according to standard protocols. All patients received an oral contrast medium. An intravenous contrast medium was administered before diagnostic CT in 359 patients.

### Vaccination

Vaccinations were administered as per manufacturer instructions in the deltoid muscle of the nondominant arm. The second vaccination was administered 21 ± 6 d after the first one. Referrals to PET/CT were according to clinical indications, independent of vaccination status.

### Visual Interpretation and Semiquantitative Analysis

All studies were reviewed by *2* experienced nuclear medicine physicians (one of whom was also a radiologist). After visual interpretation, relevant areas of increased tracer accumulation were evaluated semiquantitatively by measuring SUV_max_ with an emphasis on the posterior arm: the deltoid muscle and the axillary, supraclavicular, and cervical LNs. Increased uptake was defined as an SUV_max_ of more than 1, not compatible with anatomic or physiologic accumulation of the radiopharmaceutical. The short-axis diameter of LNs in the field of interest was recorded. LNs were defined as benign, malignant, or equivocal according to their radiologic appearance. Kidney-shaped LNs with a preserved fat center, a short-axis diameter of less than 1.0 cm, and peripheral contrast enhancement were defined as benign. O-shaped LNs that had no central fat, were larger than 1.0 cm in short-axis diameter, and showed diffuse enhancement were defined as malignant. All others were defined as equivocal.

Simultaneously increased uptake at the injection site in the arm and in ipsilateral axillary LNs was defined as the double sign (DS). Studies were interpreted without knowledge of vaccination status but with full knowledge of the clinical background.

### Statistical Analysis

Sensitivity, specificity, positive predictive value (PPV), and negative predictive value (NPV) were calculated for DS and for axillary lymphadenopathy for each of the PET tracers. Studies were considered true-positive for DS when a vaccinated patient presented with increased uptake in the ipsilateral arm and axillary LNs and true-negative when there was no vaccination before the study and no uptake in the arm and in the axilla. Vaccinated patients who showed no arm and no axillary uptake were considered to have a false-negative result. Arm and axillary uptake in nonvaccinated patients was considered a false-positive result.

For axillary lymphadenopathy, true-positive cases were defined as increased uptake in axillary lymphadenopathy in vaccinated patients, and true-negative cases were defined as lack of axillary uptake in nonvaccinated patients.

Differences between LN SUV_max_ or size with or without ipsilateral arm uptake were assessed with the Mann–Whitney *U* test. A *P* value of less than 0.05 was considered statistically significant.

## RESULTS

### ^18^F-FDG PET/CT

Between January 1, 2021, and February 15, 2021, 1,018 PET/CT studies were performed in our department, 973 of which were ^18^F-FDG PET/CT studies. Vaccination status was known for 458 patients (242 female and 216 male; age range, 8–98 y; mean ± SD, 61.3 ± 14.9 y), who made up the study group. There were 452 (99%) patients with known or suspected malignancy, and 6 (1%) were referred for the assessment of infection or inflammation. The most common clinical indications included breast cancer (*n* = 102), lymphoma and myeloma (*n* = 88), lung cancer (*n* = 86), and gastrointestinal tumors (*n* = 73). The patients’ clinical characteristics are summarized in Table 1.

**TABLE 1.** Patient Characteristics (*n* = 458)

Characteristic	Data
Age (y)	61 ± 15
Sex	
Male	216
Female	242
Indications for PET/CT	
Breast cancer	102
Lymphoma and myeloma	88
Lung cancer	86
Gastrointestinal tumor	73
Urologic and gynecologic cancer	40
Skin cancer	22
Head and neck oncology	19
Tumor of unknown origin	6
Fever of unknown origin	
Bacteremia	6
Infection or inflammation	2
Sarcoma	14

Qualitative data are number; continuous data are mean ± SD.

At the time of PET/CT, 274 of the 458 patients (60%) were vaccinated. The control group comprised the 184 patients (40%) who had not been vaccinated, 16 (3.5%) of whom had recovered from COVID-19.

### LN-Based Analysis

Axillary lymphadenopathy was present in 268 (59%) patients, 156 unilaterally and 112 bilaterally. Of these 268, 39 were vaccinated once and 141 were vaccinated twice. The LN appearance on CT was benign in 75%, malignant in 13%, and equivocal in 12% (Table 2). LN size ranged between 0.2 and 5.3 cm (mean, 0.9 ± 0.6 cm), and the SUV_max_ in the axillary LNs was 0.6–24.5 (mean, 3.5 ± 3.3). Supraclavicular LNs, ipsilateral to the axillary lymphadenopathy, with increased ^18^F-FDG activity were visualized in 26 (5.7%) patients. The sensitivity, specificity, PPV, and NPV for axillary lymphadenopathy with a benign appearance and increased activity (SUV_max_ > 1.0) were 53.7%, 84.8%, 86.5%, and 50.3%, respectively (Table 2).

**TABLE 2.** Results for ^18^F-FDG PET/CT

			Vaccinated (274 patients)		DS (54 patients)		
Parameter	Total (458 patients)	Control group (184 patients)	Once (71 patients)	Twice (203 patients)	Once (9 patients)	Twice (45 patients)	Index (%)
Mean interval from vaccination to PET/CT (d)	NA	NA	9 (range, 0–34 d)	15 (range, 0–34 d)	6 (range, 1–10 d)	9 (range, 1–21 d)	NA
Lymphadenopathy							
Patients (*n*)	268 (59%)	88 (48%)	39 (14%)	141 (51%)	9 (17%)	45 (83%)	S*, 53.7; Sp*, 84.8; PPV*, 86.5; NPV*, 50.3
LN appearance							
Benign	200 (75%)	60 (68%)	30 (77%)	110 (78%)	49 (91%)^†^		
Malignant	36 (13%)	18 (20%)	4 (10%)	14 (10%)	0^†^		
Equivocal	32 (12%)	10 (12%)	5 (13%)	17 (12%)	5 (9%)^†^		
Size (cm) in short axis	0.2–5.3	0.3–5.0	0.2–5.3^†^		0.2–5.3^†^		
SUV_max_	0.6–24.5	0.7–24.5	0.6–17.8^†^		0.9–8.6^†^		
Arm (patients [*n*])	57 (12%)	0	57 (21%)^†^		54 (100%)^†^		NA
			9 (16%)	48 (84%)	9 (17%)	45 (83%)	
DS (patients [*n*])	54 (12%)	0	54 (20%)^†^		—^†^		S, 37.2; Sp, 100; PPV, 100; NPV, 66.9
			9 (17%)	45 (83%)	—^†^		

*For unilateral axillary lymphadenopathy only, with increased uptake (SUV_max_ > 1.0) and benign appearance.

^†^Applies to both once-vaccinated and twice-vaccinated patients.

S = sensitivity; Sp = specificity; NA = not applicable.

In the control group, 88 nonvaccinated patients had unilateral axillary lymphadenopathy. LN size ranged from 0.3 to 5 cm, SUV_max_ ranged from 0.7 to 24.5, and the appearance was benign in 60 patients (68%), malignant in 18 patients (20%), and equivocal in 10 patients 12% (Table 2).

### Vaccination-Based Analysis

Of the 274 vaccinated patients, 71 (26%) were vaccinated once and 203 (74%) were vaccinated twice. The mean interval between the first vaccination and PET/CT was 9 d (range, 0–34 d). In patients who were vaccinated twice, PET/CT was performed at an average of 15 d (range, 0–34 d) after the second vaccination.

**FIGURE 1.**
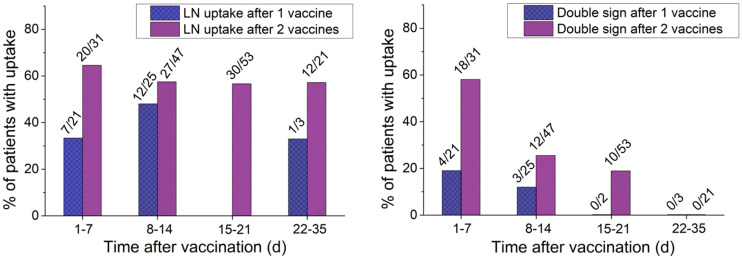
Axillary lymphadenopathy (A) and DS appearance frequency (B) as function of time after vaccination in patients vaccinated once or twice. Ratio above bars represents proportion of patients with LN or DS among vaccinated patients who underwent PET/CT at same time.

Increased uptake in axillary lymphadenopathy was observed in 180 of the 274 patients (66%): 39 (55%) of the 71 vaccinated once and (after the second vaccination) 141 (69%) of the 203 vaccinated twice. LN size ranged from 0.2 to 5.3 cm (mean, 0.8 ± 0.6 cm), and SUV_max_ ranged from 0.6 to 17.8 (mean, 3.3 ± 2.7). CT appearance and SUVs are summarized in Table 2. There was no significant difference in SUV_max_ between the first and second vaccinations. SUV_max_ in axillary lymphadenopathy decreased over time. No increased ^18^F-FDG uptake was observed 22 and 32 d after the first and the second vaccinations, respectively. Figure 1A shows the frequency of increased LN activity as a function of time after vaccination for patients who received 1 or 2 vaccinations. The frequency of increased activity was higher after the second vaccination and remained stable up to 32 d after vaccination.

Increased uptake at the vaccination site (posterior arm/deltoid muscle) was visualized in 57 patients (12%) (left, 41; right, 16), with an SUV_max_ of 1.8 ± 0.7 (range, 0.9–4.4). There was focal uptake at the vaccination site in 9 (16%) of the 57 patients after the first vaccination and in 48 (84%) after the second vaccination. CT showed mild subcutaneous fat stranding in 18 cases and no significant morphologic changes in 39 others. There was no increased uptake in the posterior arm/deltoid muscle in the control group.

### DS-Based Analysis

**FIGURE 2.**
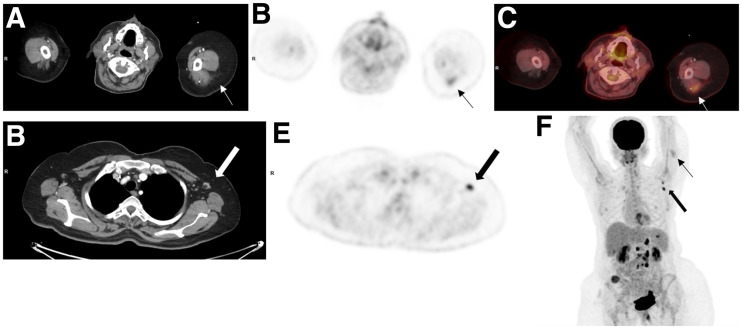
A 52-y-old woman with gastrointestinal tumor was referred for routine ^18^F-FDG PET/CT follow-up study. Study was performed 2 d after second COVID-19 vaccination. Selected transaxial CT (A and D) and PET (B and E) slices at level of posterior arm uptake and axillary LNs, fused image (C) at level of posterior arm uptake, and maximum-intensity projection (F) demonstrate moderate-intensity uptake in left posterior arm (thin arrows) (SUV_max_, 3.6) and high-grade activity in left axillary nodes measuring 1.0 cm in short axis with benign appearance (thick arrows) (SUV_max_, 7.1). Maximum-intensity projection also shows high-grade ^18^F-FDG activity in retroperitoneal nodes and multiple implants.

In 54 (20%) of the 274 vaccinated patients and 95% of the patients with increased arm uptake, there was DS (Fig. 2). DS was observed in 9 of the 54 patients (17%) after the first vaccination and in 45 (83%) after the second. The LN short-axis diameter was 0.2–5.3 cm (mean ± SD, 0.8 ± 0.7 cm), and SUV_max_ was 0.9–8.6 (mean ± SD, 3.1 ± 2.1). Among these 54 patients, 49 had nodes with a benign appearance, and in 5 patients the nodes were defined as equivocal. In 3 of the 49 patients, there were LNs with a malignant appearance adjacent to benign-appearing LNs, including a patient with lymphoma, a patient with ipsilateral breast cancer, and a patient with metastatic lung cancer.

The mean interval between the first and second vaccinations and DS was 6 and 9 d, respectively, compared with a mean of 9 and 15 d, respectively, for the entire lymphadenopathy group. DS frequency as a function of the time after vaccination in patients who received 1 or 2 vaccination doses is depicted in Figure 1B.

The highest frequency of DS was observed during the first week after the first or second vaccination (19% and 58%, respectively), with a lower frequency on PET/CT studies performed during the second week (12% and 25%, respectively) and third week (0% and 19%, respectively) after immunization. Arm uptake and DS were seen up to 10 d after the first vaccination and up to 21 d after the second. The sensitivity, specificity, PPV, and NPV of DS were 37.2%, 100%, 100%, and 66.9%, respectively.

There was no significant correlation between intensity of uptake in the arm and intensity of uptake in LNs.

There was no significant difference in LN size between the DS group (0.2–5.3 cm) and the non-DS group (0.3–5.0 cm) (*P* = 0.74). However, SUV_max_ was significantly higher in the DS group (*P =* 0.001).

### PET/CT with Tracers Other Than ^18^F-FDG

There were 31 PSMA PET/CT studies (2.4%; 26 ^68^Ga-PSMA and 5 ^18^F-PSMA) and 14 ^68^Ga-DOTATATE PET/CT studies (1.1%). A single ^18^F-FDOPA study was excluded. Vaccination data, axillary lymphadenopathy prevalence and characteristics, and DS are presented in Tables 3 and 4. Lymphadenopathy was present on PSMA studies in 26 patients (84%), 24 (92%) of whom had a benign appearance on CT, and was associated with DS in 4 patients (13%) (Supplemental Fig. 1; supplemental materials are available at http://jnm.snmjournals.org). Lymphadenopathy was present on ^68^Ga-DOTATATE studies in 12 patients (86%), all with a benign CT appearance, and DS was observed in 3 patients (21%) (Supplemental Fig. 2). The sensitivity, specificity, PPV, and NPV of DS were 44.4%, 100%, 100%, and 37%, respectively, for PSMA and 75%, 100%, 100%, and 50%, respectively, for ^68^Ga-DOTATATE.

**TABLE 3.** Results for PSMA PET/CT

			Vaccinated (28 patients)		DS (4 patients)	
Parameter	Total (31 patients)	Control group (3 patients)	Once (3 patients)	Twice (25 patients)	Once (0 patients)	Twice (4 patients)	Index (%)
Mean interval from vaccination to PET/CT (d)	NA	NA	10 (range, 6–18 d)	14 (range, 0–24 d)	NA	12 (range, 1–19 d)	NA
Lymphadenopathy							
Patients (*n*)	26 (84%)	3 (10%)	3 (11%)	20 (71%)	0	4 (100%)	S*, 61.5; Sp*, 0; PPV*, 80; NPV*, 0
LN appearance							
Benign	24 (92%)	3 (100%)	3 (100%)	18 (90%)	3 (75%)^†^		
Malignant	0	0	0	0	0^†^		
Equivocal	2 (8%)	0	0	2 (10%)	1 (25%)^†^		
Size (cm) in short axis	0.3–1.6	0.3–0.6	0.4–1.6^†^		0.7–1.5^†^		
SUV_max_	1.1–3.1	1.1–2	1.3–3.1^†^		1.4–3.1^†^		
Arm (patients [*n*])	4 (13%)	0	4 (14%)^†^		4 (100%)^†^		NA
			0 (0%)	4 (100%)	0 (0%)	4 (100%)	
DS (patients [*n*])	4 (13%)	0	4 (14%)^†^		—^†^		S, 44.4; Sp, 100; PPV, 100; NPV, 37.5
			0	4 (100%)	—^†^		

*For unilateral axillary lymphadenopathy only, with increased uptake (SUV_max_ > 1.0) and benign appearance.

^†^Applies to both once-vaccinated and twice-vaccinated patients.

S = sensitivity; Sp = specificity; NA = not applicable.

**TABLE 4.** Results for ^68^Ga-DOTATATE

			Vaccinated (13 patients)		DS (3 patients)		
Parameter	Total (14 patients)	Control group (1 patients)	Once (2 patients)	Twice (11 patients)	Once (1 patients)	Twice (2 patients)	Index (%)
Mean interval from vaccination to PET/CT (d)	NA	NA	6 (range, 3–8)	14 (range, 2–21)	3	4 (range, 2–5)	NA
Lymphadenopathy							
Patients (*n*)	12 (86%)	0	2 (15%)	10 (77%)	1 (33%)	2 (67%)	S*, 87.5; Sp*, 100; PPV*, 100; NPV*, 50^‡^
LN appearance							
Benign	12 (100%)	N/A	2 (100%)	10 (100%)	3 (100%)^†^		
Malignant	0	N/A	0	0	0^†^		
Equivocal	0	N/A	0	0	0^†^		
Size (cm) in short axis	0.5–1.1	N/A	0.5–1.1^†^		0.5–0.9^†^		
SUV_max_	1–3.5	N/A	1–3.5^†^		1.6–3.5^†^		
Arm (patients [*n*])	3 (21%)	0	3 (23%)^†^		3 (100%)^†^		NA
			1 (33%)	2 (67%)	1 (33%)	2 (67%)	
DS (patients [*n*])	3 (21%)	0	3 (23%)^†^		—^†^		S, 75; Sp, 100; PPV, 100; NPV, 50
			1 (33%)	2 (67%)	—^†^		

*For unilateral axillary lymphadenopathy only, with increased uptake (SUV_max_ > 1.0) and benign appearance.

^†^Applies to both once-vaccinated and twice-vaccinated patients.

^‡^Control group, only 1 patient.

S = sensitivity; Sp = specificity; NA = not applicable.

## DISCUSSION

The aim of present study was to identify the typical pattern on PET/CT studies after COVID-19 vaccination in order to minimize its influence on the routine workflow. We have identified DS on PET/CT studies with various radiotracers, showing increased uptake at the vaccination site and in ipsilateral axillary LNs. DS occurred with 100% PPV and specificity in up to a fifth of the vaccinated patients. When present, this highly specific imaging pattern, first described here (to our knowledge), enables avoidance of false disease upstaging or further unnecessary evaluation. Eifer et al. (*11*) showed increased uptake in the deltoid muscle and in axillary lymphadenopathy, with slightly different results for ^18^F-FDG and ^68^Ga-DOTATATE (Table 5) and lack of PSMA uptake, probably because of population heterogeneity. Interestingly, a similar pattern has been described for ^18^F-FDG PET/CT studies after influenza vaccination, with a prevalence of 5.1%–25% (*13*–*16*).

**TABLE 5.** Comparison of PET/CT Postvaccination Studies

			Days of increased lymphadenopathy uptake		
Study	Tracer	Vaccinated patients (*n*)	After first or only vaccination	After second vaccination	Increased uptake in deltoid muscle
Orevi (current study)	^18^F-FDG, ^68^Ga-DOTATATE, PSMA	503	≤22	≤32	^18^F-FDG, 20%; ^68^Ga-DOTATATE, 21%; PSMA, 13%
Cohen (*9*)	^18^F-FDG	728	∼13	∼20	NA
Eshet (*10*)	^18^F-FDG	169	NA	≤70	NA
Eifer (*11*)	^18^F-FDG, ^68^Ga-DOTATATE, PSMA	426	NA	NA	^18^F-FDG, 26%; ^68^Ga-DOTATATE, 9%; PSMA 0%
Bernstine (*12*)	^18^F-FDG	650	22	22	NA

NA = not applicable.

There was no increased uptake after third vaccination.

Stand-alone axillary lymphadenopathy can be challenging in cases of lymphoma, breast cancer, melanoma, and other cutaneous malignancies. In the present study, we have demonstrated ^18^F-FDG–avid lymphadenopathy in 59% of patients after vaccination for COVID-19, compared with 45%–46% in recent publications (*9*,*10*). The frequency of ^18^F-FDG–avid lymphadenopathy was higher after the second vaccination than after the first—69% versus 55%, respectively, in the present study, compared with 53.9% and 36.4% (*9*), respectively, and 43.3% and 14.5% (*12*), respectively, in recent publications. The prevalence of lymphadenopathy with a benign CT appearance and low-grade ^18^F-FDG accumulation was also similar, at 77% in the present study, compared with 80.1% in a previous study (*9*). This percentage is significantly higher than that for axillary lymphadenopathy after H1V1 influenza A virus vaccination (*15*), which was observed in 29.3% (*13*,*15*), or after papilloma virus vaccination (*17*), probably because of the high immunogenicity of the COVID-19 vaccine.

The mechanism of ^18^F-FDG uptake has been addressed by Eifer et al. (*11*). A strong inverse association was demonstrated between axillary LN uptake, patient age, and immune status, with avid uptake in 53% of immunocompetent patients, compared with 33% of immunocompromised patients. Deltoid uptake was associated with the time interval from the vaccine and with the number of vaccinations. The authors suggested that the activity in LNs is associated with immune system activation and that deltoid activity has an inflammatory etiology or is due to trauma induced by the injection (*11*). The mechanism of PSMA uptake in lymphadenopathy is likely mediated by PSMA expression on immune cells. A non–PSMA-related mechanism, similar to the accumulation in salivary glands, has also been suggested (*18*,*19*). ^68^Ga-DOTATATE uptake in lymphadenopathy is based on the expression of somatostatin receptors 1 and 2 on monocytes and macrophages and its regulatory role in interactions with the immune system (*20*).

The typical response to vaccination is restricted to the regional draining LNs, consistent with axillary nodes when the injection is administered in the proximal arm. Increased tracer accumulation in supraclavicular lymphadenopathy, representing the next lymphatic drainage station, was observed in 5.7% in the present study.

In the present study, increased lymphadenopathy uptake and DS was observed from day 1 after vaccination up to 22 and 32 d, respectively, after the first vaccination and 10 and 21 d, respectively, after the second vaccination. Cohen et al. have observed more significant ^18^F-FDG–avid lymphadenopathy from day 5 up to day 13 after the first vaccination and significantly lower uptake after day 20 after booster vaccination (*9*). Eshet et al. showed avid axillary lymphadenopathy 7–10 wk after the second vaccination (*10*). All studies have reported regression in ^18^F-FDG activity in lymphadenopathy and at the injection site. Table 5 summarizes the main PET/CT findings after COVID-19 vaccination in recent publications (*9*–*12*).

In contrast to a few published case reports showing a systemic inflammatory response syndrome after COVID-19 vaccination (*21*), no systemic findings were noted in our cohort or in other large cohorts. These differences are consistent with the published vaccination-related reaction data (*22*,*23*).

**FIGURE 3.**
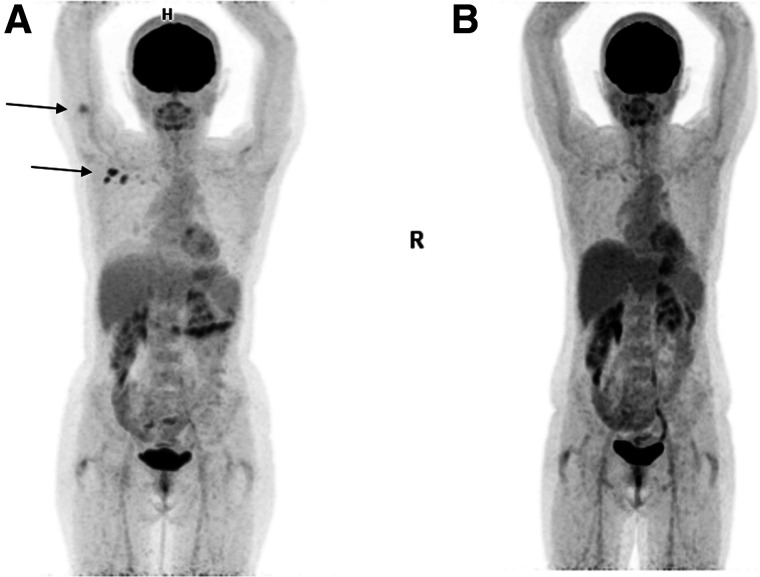
A 57-y-old woman with vulvar melanoma was referred for ^18^F-FDG PET/CT for staging (A) and 53 d later for evaluation of treatment response (B). Increased ^18^F-FDG uptake seen in right arm and in ipsilateral axilla (arrows) on baseline PET maximum-intensity projection (A) has completely resolved on second study.

Limitations of the present study are due to its retrospective nature. In addition, for obvious reasons, none of LNs were biopsied. Positive studies were not repeated to follow up the lymphadenopathy or the arm uptake. One patient returned for evaluation of treatment response 53 d after the first study and showed complete resolution of all previously visualized findings (Fig. 3).

PSMA PET/CT studies with ^68^Ga and ^18^F, both routinely used for the assessment of patients with prostate cancer, were analyzed together because of the relatively small number of these studies in the present cohort. Patients with diseases that have a predilection to cause axillary lymphadenopathy, such as melanoma, breast cancer, and lymphoma, were not excluded, reflecting the routine workflow. In these cases, the radiologic appearance of the lymphadenopathy plays a crucial role. In most cases, benign and malignant LNs can be separated, with only 8% defined as equivocal in the present study and 14.8% in another study (*9*).

Familiarity with postvaccination patterns on PET/CT is important for the interpreting, as well as the referring, physicians. To prevent erroneous interpretation of postvaccination lymphadenopathy, it has been previously suggested that PET/CT be postponed until recovery from postimmunization lymphadenopathy. However, lymphadenopathy may persist for up to 70 d (*11*) after vaccination, and such prolonged delays should be avoided, especially in cancer patients. We recommend adding to the routine patient questionnaire the dates and locations of vaccination and any history of COVID-19 infection. It is also advisable to actively look for DS in addition to routinely interpreting the LN appearance. Awareness of the different patterns may help prevent false-positive interpretations of PET/CT studies.

## CONCLUSION

COVID-19 vaccination frequently causes axillary radiotracer-avid lymphadenopathy and postinjection uptake in the arm. DS, observed in the present study in 20% of postvaccination patients, is highly specific for postvaccination lymphadenopathy and can reduce misinterpretation of PET/CT and the consequences of that misinterpretation. The present study included a large patient population but was a single-center study with only 1 type of vaccine. Further similar studies with other types of COVID-19 vaccine are needed.

## DISCLOSURE

No potential conflict of interest relevant to this article was reported.

## Notes

KEY POINTS **QUESTION:** How does COVID-19 vaccine lymphadenopathy challenge the interpretation of PET/CT studies? **PERTINENT FINDINGS:** Post–COVID-19 vaccination PET/CT studies showed stand-alone axillary lymphadenopathy in 68% of patients. A fifth of the patients had simultaneous tracer accumulation at the injection site in the arm and in ipsilateral axillary LNs. **IMPLICATIONS FOR PATIENT CARE:** The finding of simultaneous tracer accumulation at the injection site in the arm and in ipsilateral axillary LNs was highly specific for postvaccination lymphadenopathy, and awareness can prevent misinterpretation, unnecessary further evaluation, and its consequences.
